# Uncovering a Rare Case of Hepatic Round Ligament Epigastric Hernia

**DOI:** 10.7759/cureus.57553

**Published:** 2024-04-03

**Authors:** Natalia M Barron-Cervantes, Alejandro Martinez-Esteban, Eduardo Villegas-Tovar, Fabiola M Nuccio-Giordano, Alejandro D. G. Gidi

**Affiliations:** 1 General and Gastrointestinal Surgery Service, Fundación Clínica Médica Sur, Mexico City, MEX; 2 General Surgery, Fundación Clínica Médica Sur, Mexico City, MEX; 3 Anesthesia, Fundación Clínica Médica Sur, Mexico City, MEX; 4 General and Gastrointestinal Surgery, Angeles Health System, Mexico City, MEX

**Keywords:** laparoscopic hernia repair, mesh repair, epigastric hernias, round ligament hernia, abdominal hernias

## Abstract

Abdominal wall hernias are one of the most common surgical diseases present in both males and females nowadays. However, with only a few cases reported in the literature, hepatic round ligament hernias are a rare clinical manifestation. This case shows how a common symptom such as epigastric pain can be associated with this rare condition. In general, abdominal computed tomography (CT) images are the choice of study to evaluate complications and the involvement of different intestinal sections. Some laboratory tests can be performed to suspect intestinal ischemia secondary to strangulated hernias. Primary repair utilizing mesh is the preferred surgical treatment. This procedure can be performed through laparoscopic or open technique, depending on the surgeon's skills and patient preference.

## Introduction

Abdominal wall hernias are a really common disease present in both males and females nowadays, with its surgical repair being the second most common abdominopelvic operation in the United States, after cesarean delivery [1]. They are usually associated with increased intra-abdominal pressure or prior surgeries [1,2]. According to their severity classification, they can be classified as uncomplicated or reducible and complicated or non-reducible. These are also divided into strangulated and incarcerated. It is important to mention that the imaging study of choice for a suspected complicated hernia is an abdominal intravenous (IV) contrast computed tomography (CT) scan. In this study, it is easier to identify the defect in the abdominal wall, the hernia sac, and the content, which are the three main components of an abdominal hernia [3]. The content is mostly omental fat, and on some occasions, it can be found in gastrointestinal content, such as intestine, solid organs, or even ligaments, as in this case. Multi-detector row CT offers a precise anatomical depiction of the abdominal wall, which also helps clinicians distinguish hernias from other abdominal masses such as tumors, hematomas, or abscesses and even identify complications presented. Emphasizing that abdominal CT is crucial for accurate diagnosis and informed clinical decision-making is critical [3].

Because of the high risk of developing complications, most abdominal wall hernias undergo surgical treatment even when asymptomatic. However, it is important to mention that post-surgical complications are also common and include hernia recurrence, infected and non-infected fluid collections, and complications related to prosthetic material [3]. Intestinal ischemia is a complication that can occur in strangulated hernias and is associated with high mortality due mainly to a delay in diagnosis. Although angiotomography is an appropriate imaging study to evaluate it, there are serological markers that help clinicians suspect it and thus be able to address it in a more complete and timely manner. Some of these markers include lactate, lactic dehydrogenase (LDH), D-dimer, procalcitonin, and others mentioned in the Discussion [4].

We present the case of a 32-year-old female patient who presented with progressive epigastric pain (9/10) that presented after lifting weights at the local gym. This was presented in a first-level private surgical center in Mexico City. This case is presented to further expand the knowledge about round ligament hernias, as well as highlight the importance of an early approach to improve morbidity in these cases.

## Case presentation

A previously healthy 32-year-old female presented to the emergency department (ED) with progressive epigastric pain (9/10) that presented after lifting weights at the local gym. She mentioned that the pain started immediately after having done the physical activity two hours prior with no radiation of the pain. It was accompanied by nausea, but no emesis presented, and increased volume in the epigastric region. Because of the clinical presentation, she self-medicated with butylhyoscine 10 mg per oral (PO) in a single dose and acetaminophen 1 gram PO in a single dose. In the absence of any amelioration in her clinical state, she elected to seek evaluation at the ED. Upon physical examination, an epigastric mass of approximately 2 cm in diameter was found. It did not show any changes in color or temperature on the surface, but it presented a stony consistency, painful on mid-superficial palpation, fixed to deep planes, without reduction to manual maneuvers. Normal peristalsis sounds were noted, and no abdominal distension or data suggestive of peritoneal irritation was recognized. During her stay at the ED, comprehensive laboratory tests were conducted (Table 1), revealing deep thrombocytopenia, mild hypokalemia, and no other abnormalities. It is important to emphasize that all ischemia markers were negative, and there was no data suggestive of infection.

**Table 1:** Initial laboratory results Relevant laboratory tests include CBC, BCT, coagulation times, and ischemia markers. CBC: complete blood count, BCT: blood chemistry test, BUN: blood urea nitrogen, PT: prothrombin time, PTT: partial thromboplastin time, INR: international normalized ratio, LDH: lactic dehydrogenase

Parameter	Value	Reference values
Hemoglobin	16.4 g/dL	12-18 g/dL
Hematocrit	49.3%	36%-50%
Platelets	25.103 × 10^3^/uL	150-450 × 10^3^/uL
Leukocyte count	9.8 × 10^3^/uL	4.5-11 × 10^3^/uL
Serum sodium	137 mmol/L	135-145 mmol/L
Serum potassium	3.4 mmol/L	3.6-5.2 mmol/L
Serum calcium	9.1 mmol/L	8.6-10.3 mmol/L
Serum glucose	98 mg/dL	72-99 mg/dL
Serum creatinine	0.8 mg/dL	0.31-1.00 mg/dL
BUN	10 mg/dL	7-22 mg/dL
PT	11.3 seconds	11-13.5 seconds
PTT	27.4 seconds	25-35 seconds
Thromboplastin time	28.5 seconds	24.5-32.0 seconds
INR	0.9	<1.1
Lactate	<2 mmol/L	<2 mmol/L
D-dimer	270 μg/L	0.00-499.00 μg/L
LDH	152 U/L	140-280 U/L
Procalcitonin	<0.02 ng/mL	0.00-0.06 ng/mL

**Figure 1:**
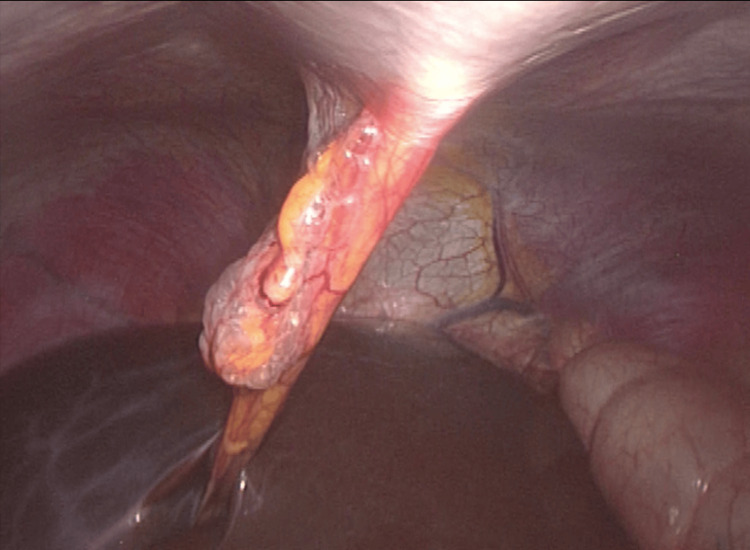
Herniation of the round ligament Herniation of the round ligament with a wall defect of 2 cm in greatest diameter.

Because of the clinical presentation, an abdominal CT scan was performed, which reported an abdominal wall defect of 2 × 2 cm, located 3 cm above the umbilical scar with herniation of the hepatic round ligament, as well as omental fat, presenting fat striation compatible with complicated incarcerated wall herniation. The imaging study was performed at another outpatient clinic, and the patient only presented the final report because of economic factors. At the clinic where the patient presented, a decision was made to not repeat the CT scan. A preoperative diagnosis of incarcerated abdominal wall hernia with hepatic round ligament content was made, and minimal invasive surgical resolution was decided. Under balanced general anesthesia, diagnostic laparoscopy was performed, and herniation of the round ligament with a wall defect of 2 cm in greatest diameter was found (Figure 1).

**Figure 2:**
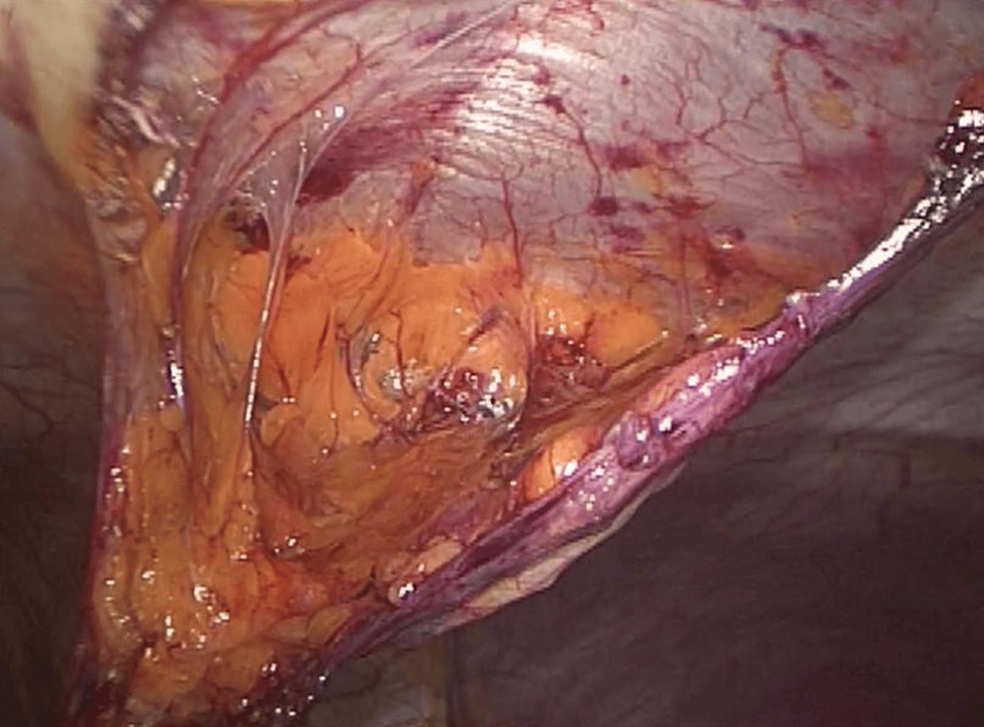
Round ligament dissection The round ligament was dissected at the level of the defect, reduced, and subsequently cut.

**Figure 3:**
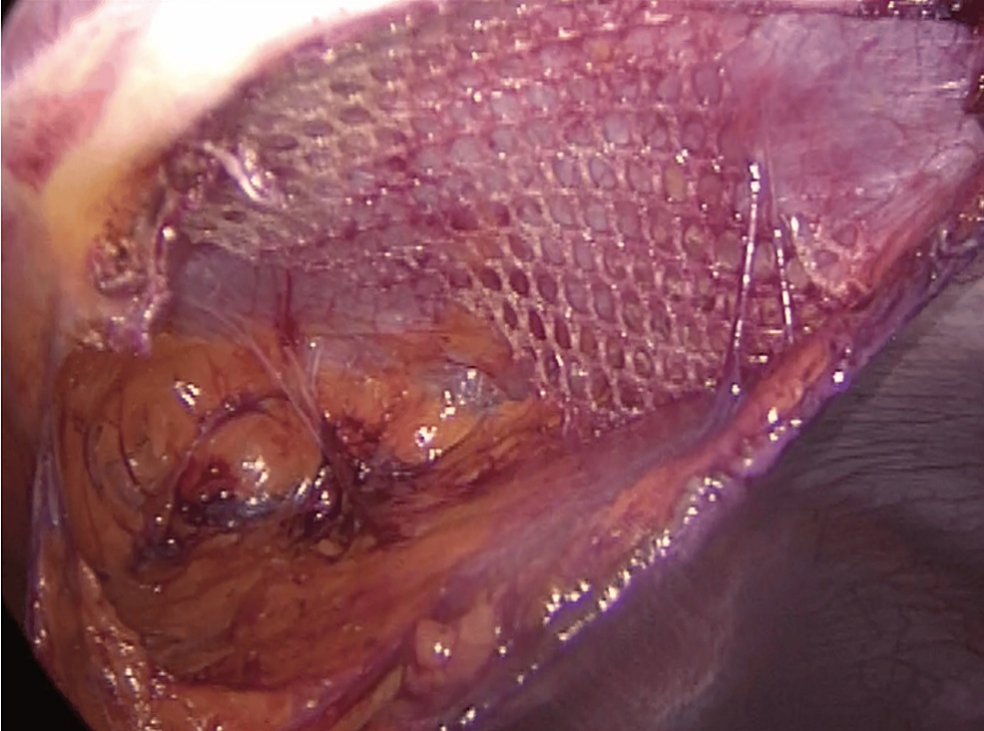
Mesh repair Self-adherent mesh was used to repair the abdominal wall defect.

**Figure 4:**
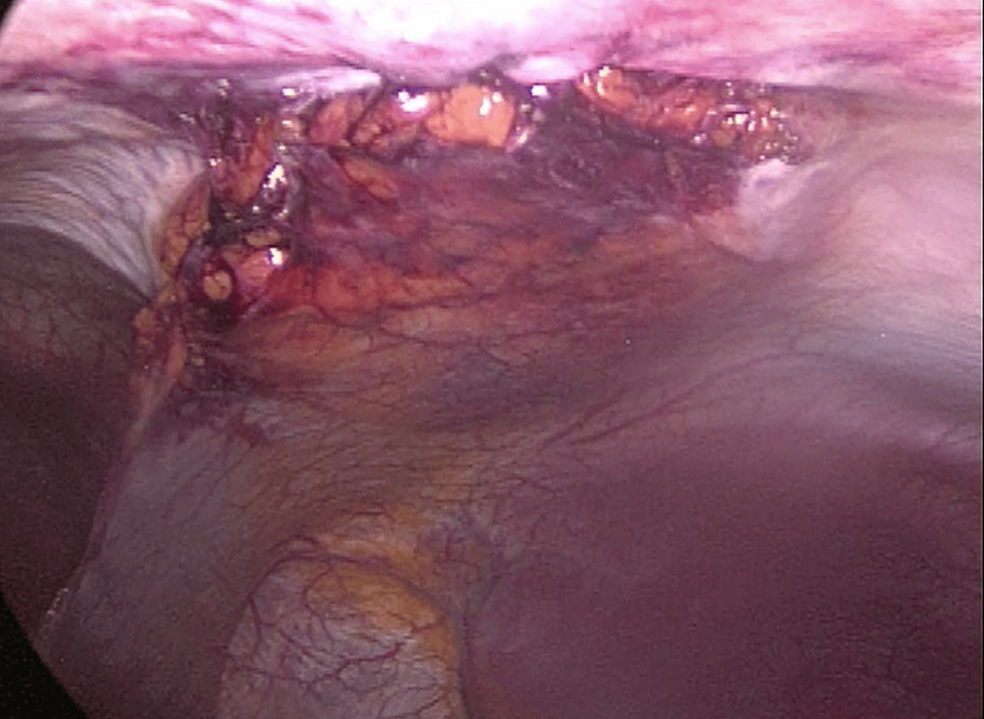
Peritoneum closure Peritoneal closure after hernia repair.

The round ligament was dissected at the level of the defect, reduced, and subsequently cut (Figure 2). A self-adherent polypropylene mesh was placed (Figure 3), and the peritoneum was closed (Figure 4). The postoperative evolution was satisfactory without any complications, and the patient was discharged the next postoperative day.

The final histopathological study reported an elongated and irregular specimen measuring 9.3 × 1.5 × 1 cm with an adipose appearance and a light gray area with a fibrous appearance, presenting a soft consistency when cut. A final diagnosis of fibroadipose tissue compatible with hernia sac contents, hernial sac with fibrosis, and mild chronic inflammation of the hepatic round ligament was given. The patient presented to her follow-up appointment four weeks postoperatively with no complications and completely asymptomatic, and was discharged from the service.

## Discussion

Epigastric hernias are a rare condition with a reported prevalence of 10% in the United States [5]. Only a minority of cases present as symptomatic. They represent 1.6%-3.6% of all abdominal hernias and 0.5%-5% of all operated abdominal hernias. It presents more commonly in males, with a higher incidence in the population between 20 and 50 years [5]. The most accepted physiopathological theory explains that epigastric hernias are the result of the extra tension in the epigastric region secondary to the diaphragm attachment [5]. As previously mentioned, both of them are complicated hernias; however, the main difference between them is that incarcerated hernias are only hernias in which the content cannot be reduced but does not present any vascular involvement at the moment. Thus, this does not elevate ischemia markers, as in the case presented in this report. Strangulated hernias, on the other hand, always present with vascular involvement, which means that a certain degree of ischemia or necrosis is present at the moment when the diagnosis is made [6].

Intestinal ischemia is associated with high mortality rates, and abdominal contrast-enhanced CT scan remains a sensitive and specific tool used in most cases. However, the presence of some serological markers may guide clinical decision-making to perform one angiography CT in cases where ischemia is not as clear. A meta-analysis performed in Spain in 2023 concluded that lactate, at least in its L form, does not help with early diagnosis since, in addition to being nonspecific, its elevation occurs in advanced stages. Nevertheless, the D form of lactate along with D-dimer and procalcitonin presents as the most sensitive combination of serological markers that may indicate intestinal ischemia and allow physicians to order more specific imaging studies to confirm its diagnosis [4]. In this case, in particular, this information was really important to decide whether only a non-contrast abdominal CT scan should be performed for the patient.

An abdominal CT scan is one of the most used image studies to diagnose hernias. It not only provides accurate data about its location but also allows the physician to identify the content and if it is complicated or not and even determine the surgical approach that will be used. Epigastric hernias can be diagnosed through abdominal CT as abdominal wall defects along the xipho-umbilical line through stretching of the linea alba. In general, this type of hernia contains preperitoneal fat, vascular structures, bowel, and, occasionally, stomach [7]. The presentation of an epigastric hernia including the round ligament of the liver has been seen only in a few cases throughout the literature. According to the guidelines for the treatment of umbilical and epigastric hernias from the European Hernia Society and the American Hernia Society [8], these procedures are frequently performed throughout the world and present an expected low complication rate. The preferred method of repair is using a preperitoneal flat mesh as it is associated with a lower recurrence rate, and a laparoscopic approach is the preferred method for large and small hernias in any patient who has an increased risk of wound morbidity. Following these guidelines, all symptomatic umbilical and epigastric hernias must be repaired by open approach with a preperitoneal flat mesh unless the patient wants a laparoscopic approach or presents an increased risk of round morbidity [8]. It is important to emphasize that for a procedure to be successful over time, the most important factor is the individual ability of the surgeon, so even when the patient decides to have a laparoscopic approach, it should always be discussed with an expert in this field [9]. Although laparoscopic repair is associated with a lower recurrence rate, some factors, such as prior failed hernia repair and increased estimated blood loss, were associated with higher recurrence rates despite having a laparoscopic procedure [10].

## Conclusions

The occurrence of epigastric umbilical hernias, particularly those involving the round ligament of the liver, represents a rare clinical phenomenon, with symptomatic presentations being even more infrequent. Prompt diagnosis assumes great importance, given the potential for severe complications such as intestinal ischemia, which carries a notable mortality risk. Consequently, employing serological markers to guide appropriate imaging modalities becomes imperative to effectively exclude such complications. This case underscores the distinctive nature of epigastric umbilical hernias, particularly when characterized by the presence of solid contents within the hernia sac. The clinical outcome following surgical intervention in this instance was favorable, underscoring the significance of proficient surgical technique coupled with timely diagnosis, which facilitated early surgical intervention.
